# High Fat Diet Modulates *Trypanosoma cruzi* Infection Associated Myocarditis

**DOI:** 10.1371/journal.pntd.0003118

**Published:** 2014-10-02

**Authors:** Fnu Nagajyothi, Louis M. Weiss, Dazhi Zhao, Wade Koba, Linda A. Jelicks, Min-Hui Cui, Stephen M. Factor, Philipp E. Scherer, Herbert B. Tanowitz

**Affiliations:** 1 Department of Pathology, Albert Einstein College of Medicine, Bronx, New York, New York, United States of America; 2 Department of Medicine, Albert Einstein College of Medicine, Bronx, New York, New York, United States of America; 3 Department of Radiology, Albert Einstein College of Medicine, Bronx, New York, New York, United States of America; 4 The Gruss Magnetic Resonance Research Center, Albert Einstein College of Medicine, Bronx, New York, New York, United States of America; 5 Department of Physiology and Biophysics, Albert Einstein College of Medicine, Bronx, New York, New York, United States of America; 6 Departments of Internal Medicine and Cell Biology and the Touchstone Diabetes Center, University of Texas, Southwestern Medical Center, Dallas, Texas, Unites States of America; Universidade Federal de Minas Gerais, Brazil

## Abstract

**Background:**

*Trypanosoma cruzi*, the causative agent of Chagas disease, has high affinity for lipoproteins and adipose tissue. Infection results in myocarditis, fat loss and alterations in lipid homeostasis. This study was aimed at analyzing the effect of high fat diet (HFD) on regulating acute *T. cruzi* infection-induced myocarditis and to evaluate the effect of HFD on lipid metabolism in adipose tissue and heart during acute *T. cruzi* infection.

**Methodology/Principal Findings:**

CD1 mice were infected with *T. cruzi* (Brazil strain) and fed either a regular control diet (RD) or HFD for 35 days following infection. Serum lipid profile, tissue cholesterol levels, blood parasitemia, and tissue parasite load were analyzed to evaluate the effect of diet on infection. MicroPET and MRI analysis were performed to examine the morphological and functional status of the heart during acute infection. qPCR and immunoblot analysis were carried out to analyze the effect of diet on the genes involved in the host lipid metabolism during infection. Oil red O staining of the adipose tissue demonstrated reduced lipolysis in HFD compared to RD fed mice. HFD reduced mortality, parasitemia and cardiac parasite load, but increased parasite load in adipocytes. HFD decreased lipolysis during acute infection. Both qPCR and protein analysis demonstrated alterations in lipid metabolic pathways in adipose tissue and heart in RD fed mice, which were further modulated by HFD. Both microPET and MRI analyses demonstrated changes in infected RD murine hearts which were ameliorated by HFD.

**Conclusion/Significance:**

These studies indicate that Chagasic cardiomyopathy is associated with a cardiac lipidpathy and that both cardiac lipotoxicity and adipose tissue play a role in the pathogenesis of Chagas disease. HFD protected mice from *T. cruzi* infection-induced myocardial damage most likely due to the effects of HFD on both adipogenesis and *T. cruzi* infection-induced cardiac lipidopathy.

## Author Summary

Infection with *Trypanosoma cruzi*, the etiologic agent in Chagas disease, may result in heart disease. There has been an increase in obesity, diabetes, hypertension and ischemic cardiovascular disease in endemic areas. Previously, we demonstrated that adipose tissue is an early target and a reservoir for *T. cruzi*. *T. cruzi* has high affinity for lipoproteins, and that infected tissues there is an increase in intra-cellular cholesterol levels. It is likely that adipocytes and lipoproteins play a key role in the pathogenesis of Chagas disease. The role of host lipids in the pathogenesis of Chagas disease is understudied. Diet plays a major role in the regulation of systemic and whole body lipid levels including adipogenesis and lipogenesis. We report, for the first time, the effect of diet on myocardial inflammation and damage observed during acute *T. cruzi* infection and provide data on the role of parasite associated LDL/HDL in the regulation of systemic lipid homeostasis in white adipose tissue (WAT) and in the heart. Interestingly, we demonstrate that a high fat diet protects mice from the consequences of infection-induced myocardial damage through effects on adipogenesis in adipose tissue and reduced cardiac lipidopathy.

## Introduction

Chagas disease, caused by the parasite *Trypanosoma cruzi*, is classified by WHO as a neglected tropical disease and is a major cause of morbidity and mortality in Latin America [1]. Globalization has led to the increased recognition of this infection among immigrants from Latin America in non-endemic countries [2]. It has been estimated that 18 to 20 million people have Chagas disease [2]. Symptoms of infection are varied, but include heart disease and megasyndromes of the gastrointestinal tract. Chagas disease has acute, indeterminate and chronic phases. Acute systemic infection is often asymptomatic, but, in those that are symptomatic the disease is characterized by myocarditis and/or meningoencephalitis [1]–[3]. *T. cruzi* infection causes an intense systemic pro-inflammatory response in many organs including the heart. Following infection the majority of patients develop an asymptomatic latent infection termed the indeterminate (or latent) stage of infection. As many as thirty percent of infected individuals may progress to chronic disease characterized by cardiomyopathy and/or mega syndromes. Myocardial dysfunction is associated with extensive remodeling caused by the initial infection and ensuing fibrosis [4].

The low density lipoprotein receptor (LDLr) is involved in LDL internalization and regulation of cholesterol homeostasis [5]. We have demonstrated that *T. cruzi* utilizes LDLr to invade host cells and that LDLr likely plays an important role in the pathogenesis of Chagas disease [6]. *T. cruzi* has high affinity for LDL and HDL and the rate of invasion increases in the presence of lipoproteins [7]. *T. cruzi* primarily targets lipid rich adipose tissue as their reservoir and causes lipolysis during acute infection [8], [9]. Altered serum triglyceride and cholesterol levels are associated with acute infection [6].

The role of host lipids in the pathogenesis of Chagas disease is understudied. Diet plays a major role in the regulation of systemic and whole body lipid levels including adipogenesis and lipogenesis [10]. Recent changes in diet, underlying the well-recognized obesity epidemic, in regions endemic for Chagas Disease are likely to have significant effects on the interaction of this parasite with its human host. Herein, we report, for the first time, the effect of diet on myocardial inflammation and damage seen during acute *T. cruzi* infection. We also provide data on the role of parasite associated LDL/HDL in the regulation of systemic lipid homeostasis in white adipose tissue (WAT) and in the heart.

## Materials and Methods

### Ethical approval

All animal experimental protocols were approved by the Institutional Animal Care and Use Committees (IACUC) of Albert Einstein College of Medicine (No. 20130202) which is adhered to the National Research Council guidelines (Guide for the Care and Use of Laboratory Animals: Eight Edition, Washington, DC: The National Academies Press, 2011).

### Experimental animal model

The Brazil strain of *T. cruzi* was maintained by passage in C3H/Hej mice (Jackson Laboratories, Bar Harbor, ME). Male CD-1 mice (Jackson Laboratories) were infected intraperitoneally (i.p.) at 8–10 weeks of age with 5×10^4^ trypomastigotes of the Brazil strain [8]. Mice were maintained on a 12-hour light/dark cycle. Mice, starting at the day of infection, were randomly divided into two groups (n = 15 per group) and fed on either high fat diet (HFD; 60% fat) or Regular diet (RD, 10% fat) (D12492 or D12450 Research Diets, Inc., New Brunswick, NJ). Uninfected mice were fed on either HFD (n = 15) or RD (n = 15) and used as respective controls in all the experiments. For each replication of this experiment the same numbers of mice were used in all groups.

Plasma samples were obtained from 75 µl of blood collected from the orbital venous sinus (using isoflurane anesthesia) at 10, 15, 20, 25 and 30 days post infection (dpi). Parasitemia was evaluated by counting in a Neubauer hemocytometer as described previously [8]. Thirty five days after infection the mice were euthanized and heart and epididymal white adipose tissues (WAT) were harvested for analysis. At this time-point there was no peripheral parasitemia and mice appeared normal.

### Analysis of lipid profile

Colorimetric assays were performed using colorimetric assay kit for non-esterified fatty acid (NEFA from Cell Biolabs, Inc. CA), triglyceride (TG from Cayman Chemicals, MI), and low density lipoprotein (LDL), high density lipoprotein (HDL) and total cholesterol (TC) (Enzychrom (E2HL-100), Bioassay system, CA) in serum samples^6^. Cholesterol levels were quantified in the hearts and WATs of mice at d35pi using a colorimetric assay kit and samples were prepared and assayed following manufacturer's protocol (Total cholesterol colorimetric assay kit, Cell Biolabs Inc., CA). The lipid content of frozen WAT was quantified using Oil Red O staining. Frozen sections stained with Oil Red O were washed 3 times in isopropyl alcohol for 3 minutes each. Bound Oil Red O was eluted by incubating in isopropyl alcohol (5 mL) for 16 hours; the eluted lipid content stained with Oil Red O was then measured at 540 nm in a spectrophotometer (Shimadzu uv-1201) as previously reported [9].

### Immunoblot analysis

Tissue lysates were prepared as previously described [6]. An aliquot of each sample (40 µg protein) was subjected to a 4–15% gradient SDS-PAGE and the proteins transferred to nitrocellulose filters for immunoblot analysis. LDLr specific rabbit monoclonal antibody (1∶1000 dilution, AB52818 Abcam, Cambridge, MA), lipoprotein lipase (LPL) specific mouse monoclonal antibody (1∶1000 dilution, AB21356, Abcam), or ABCA1 specific rabbit monoclonal antibody (1∶1000 dilution, AB18180, Abcam) were used as primary antisera. Horseradish peroxidase-conjugated goat anti-mouse immunoglobulin (1∶2000 dilution, Amersham Biosciences, Piscataway, NJ) or horseradish peroxidase- conjugated goat anti-rabbit immunoglobulin (1∶5000 dilution, Amersham Biosciences) were used to detect specific protein bands (explained in Figure Legends) using a chemiluminescence system [6]. GDI (1∶10000 dilution, 71-0300, and rabbit polyclonal, Invitrogen, CA) and a secondary antibody horseradish peroxidase conjugated goat anti-rabbit (1∶2000 dilution, Amersham Biosciences) were used to normalize protein loading. Rabbit Adiponectin antibody was produced in the laboratory of Dr Scherer as described previously [8].

### Quantitative determination of parasite load in tissue

Heart and white adipose tissue were collected from mice on 35 days post-infection and stored at −80°C. A quantitative real-time polymerase chain reaction (qPCR) was used to quantify parasite load employing PCR SYBR Green Master Mix (Roche Applied Science, CT) containing MgCl_2_ employing an iQ5 LightCycler (Bio-Rad). Isolation of DNA, preparation of standard curves for host and epimastigote DNA, and qPCR analysis was performed as previously published [8]. Host 18srRNA gene was used for normalization [18S forward: 5′-AGGGTTCGATTCCCGGAGAGG-3′, reverse, 5′-CAACTTTAATATACGCTATTGG-3′].

### Polymerase chain reaction array

An RT^2^ Profiler (SA Biosciences, Valencia, CA) custom designed PCR array for mouse genes involved in LDL internalization, cholesterol metabolism, fatty acid and triglyceride metabolism, glucose metabolism and inflammatory signaling was used to analyze gene expression. Data analysis was performed normalized to the expression of 18sRNA using the ΔΔCT method according to the manufacturer's protocol (SABiosciences) and statistical analysis was performed as suggested [9].

### Immunohistochemistry and immunofluorescence analysis (IFA)

Freshly isolated tissues were fixed with phosphate-buffered formalin overnight and then embedded in paraffin wax (n = 5). Hematoxylin and eosin (H&E) staining was performed and the images were captured as previously published [8]. Four to six sections of each heart were scored blindly. For each myocardial sample, histologic evidence of myocarditis and inflammation was classified in terms of degree of degenerating cardiac muscle fibres, inflammation, fibrosis and adipocyte presence and was graded on a five point scale ranging from 0 to 4+. A zero score indicated lowest or negligible changes and 4 the most damaged state. IFA was performed on the frozen sections using anti-LDL and the images were captured as previously published [6]. The fluorescent intensities of the images were quantified using NIH-Image J program for four to six images of each heart.

### Micro-positron emission tomography (MicroPET) analysis

All mice were imaged after 3 hours of fasting. Mice were administered 300–400 uCi (12–15 MBq) in 0.1 mL normal saline, [^18^F] fluoro-2-deoxyglucose (FDG), via tail vein and imaging was started at 1 hour after injection. This period permits the tracer to be delivered throughout the body and trapped by the glycolytic pathway. Prior to FDG administration the mice were anesthetized with 1.5% isoflurane-oxygen mixture, which continued throughout the imaging portion of the procedure. After MicroPET imaging, the animals were housed in the imaging facility for ten half lives (^18^F has a half-life of 110 minutes) until they could be safely moved back to the Animal Institute for continued housing.

The mice were imaged by an Inveon Multimodality scanner (Siemens, Knoxville, TN) using its PET module. PET imaging is performed using the PET gantry, which provides a 12.7 cm axial and 10 cm transaxial active field of view. The PET scanner has no septa, and acquisitions are performed in the 3D list mode. A reconstructed full-width-half-max resolution of <1.4 mm is achievable in the center of the axial field of view. List mode acquisition of data is performed to permit dynamic re-framing for kinetic evaluation of the radiotracer uptake, where indicated. After each acquisition, data were sorted into 3D sinograms, and images were reconstructed using a two dimensional (2D)-Ordered Subset Expectation Maximization algorithm. Data were corrected for decay, dead time counting losses, random coincidences and the measured nonuniformity of detector response (i.e. normalized), but not for attenuation or scatter. Analysis was performed by using the Inveon Research Workplace 4.1 software (Siemens). All imaging studies were inspected visually in a rotating 3D projection display to identify interpretability and image artifacts. Regions of interest (ROI) were manually defined. Successive scrolling through 2D slices (each 1.2 mm thick in the axial images) permitted measurement of radioactivity within defined volumes. Corrected counts per cc within this volume divided by the counts per gram of total body mass of injected radioactivity determined the SUV. SUV_max_, the maximum value of SUV within the heart was determined. The SUVmax is the maximum value of the percent-age injected dose per gram of cardiac tissue multiplied by the body weight of each animal. The SUVmax has been validated in numerous animal and human models as a reproducible and robust measure of radioactivity in longitudinal studies.

### Magnetic resonance imaging (MRI) analysis

Cardiac gated MRI was performed on uninfected and infected mice at 26 dpi were imaged using a 9.4 T Varian Direct Drive animal magnetic resonance imaging and spectroscopic system (Agilent Technologies, Inc. Santa Clara, CA) as previously published [10]. Briefly, anesthesia was induced with 2% isoflurane in air, mice were positioned supine inside an MR compatible holder and positioned within a 35-mm ID quadrature ^1^H volume coil (Molecules2Man Imaging Co., Cleveland, OH). Body temperature was maintained at 34∼35°C using warm air with feedback from a body surface thermocouple. A respiratory sensor balloon was taped onto the abdomen. Cardiac (ECG electrodes inserted subcutaneously in front left paw and rear right paw) and respiratory signal (from sensor balloon taped to the abdomen) were continuously monitored and used for MR gating/triggering by an SA Monitoring and Gating System (Small Animal Instruments, Inc., Stony Brook, NY). Ten to fourteen 1-mm-thick slices without gap was acquired in short-axis orientation covering the entire heart using an ECG-triggered and respiratory gated multi-frame tagged cine sequence. The imaging parameters used were field of view (FOV) of 40×40 mm^2^, matrix size of 256×256, TE of 2.6 ms, TR of 5.5 ms, flip angle of 25°, number of averages of 2. The number of frames was twelve to eighteen. Data were transferred to a PC and analyzed using MATLAB-based software. Left ventricle (LV) and right ventricle (RV) dimensions in millimeters were determined from the images representing end-diastole. The left ventricular wall is the average of the anterior, posterior, lateral, and septal walls. The right ventricular internal dimension is the widest point of the right ventricular cavity.

### Statistical analysis

Immunoblot, immunofluorescence and quantification of parasite load studies were performed at least three times and representative data are presented in the figures. Lipid profile analysis and gene arrays were done in duplicates. Data were pooled and statistical analysis was performed using a Student's t-test (Microsoft Excel) as appropriate and significance of difference was determined as p values between <0.05 and <0.005.

### Accession numbers (NCBI)

Cxcl16 NM_023158.6, Stab1 NM_138672, Vldlr NM_013703, Lrp6 NM_008514, Ldlr NM_010700, Scarf1NM_001004157, Apoa1NM_009692, Apob NM_009693, Apoe NM_009696, Acaa1aNM_130864,

Acad9 NM_172678, Acad10NM_028037, Acox1NM_015729, Fabp1NM_017399, Acsbg1NM_053178,

Lipe NM_010719, Npc1NM_008720, Lcat NM_008490, Abca1NM_013454, Abcg1NM_009593, Cyp39a1NM_018887, Cyp7a1NM_007824, Hmgcr NM_008255, Insig1NM_153526, Lep NM_008493,

Ppara NM_011144, Pparg NM_011146, Adig NM_145635, Adipoq NM_009605

## Results

### HFD fed mice displayed decreased mortality, parasitemia and myocardial parasite load during acute *T. cruzi* infection

To investigate the impact of diet on the course of acute *T. cruzi* infection we initially studied CD-1 mice that were placed on either a high fat diet (HFD (20 kcal% protein, 20 kcal% carbohydrate and 60 kcal% fat)) or regular diet (RD (20 kcal% protein, 70 kcal% carbohydrate and 10 kcal% fat)) at the time of infection (i.e. on the first day of infection). HFD fat content is composed of saturated (81.5 g), monounsaturated (91.5 g) and polyunsaturated (81.5 g) fat and RD fat content is composed of saturated (9.9 g), monounsaturated (13 g) and polyunsaturated (20.7 g) fat. No significant differences in the body weight were observed between the groups at the start of infection. As time progressed, the infected RD fed mice displayed fat loss (60%), as quantified by oil red O staining [9] compared to uninfected mice and this was associated with edema on 35 days post infection (dpi) as previously described [8]. Analysis suggested that infected RD fed mice gained weight due to edema and the infected HFD fed mice mainly due to fat. In contrast, infected HFD fed mice displayed no signs of edema and had only a 20% fat loss compared to uninfected HFD fed mice. No significant difference was observed in the food take between RD-fed or HFD-fed mice during infection.

**Figure 1:**
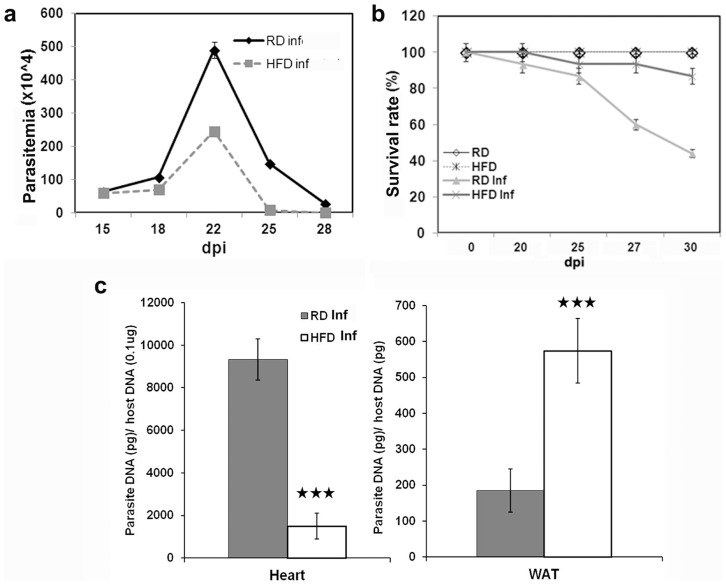
HFD modulates survival rate by decreasing parasite load in the hearts of infected mice during acute infection (n = 15). (**a**) Mice fed on HFD display a 2 fold decrease in parasitemia compared to RD fed mice. (**b**) qPCR analysis demonstrates a significant decrease (5 fold) of parasites in the hearts of HFD fed infected mice compared to the infected RD fed mice. However, WAT show no significant difference between the mice fed on HFD or RD (p≤0.005, represented by black stars). (**c**) HFD fed mice displayed a better survival (85%) compared RD fed mice (40%). Bars represent mean values of the data with Standard Error of the mean (SEM) as vertical lines.

Peak parasitemia was observed between 15 and 28 dpi in all groups. There was a twofold decrease in parasitemia in HFD fed mice compared to RD fed mice (Fig. 1a). HFD fed mice had a significantly increased survival rate (85%) compared to RD fed mice (40%) during acute infection (Fig. 1b). In addition, a higher parasite load was seen in the myocardium of RD fed mice as determined by qPCR (Fig. 1c). However increased parasite loads were detected in white adipose tissue (WAT) of HFD compared to RD fed mice (Fig. 1c).

We next investigated the effect of *T. cruzi* infection on the rate of mortality and parasitemia in mice that were placed on a HFD or RD for 30 days before infection (i.e. diet pre-fed mice) and were then continued on the respective diets for 35 dpi. HFD pre-fed mice had a 92% survival compared with a 40% survival of the RD pre fed mice due to acute infection. These data demonstrate that HFD has a protective effect on the mortality seen during acute *T. cruzi* infection in this murine model.

Similar to what was seen in mice started on HFD and RD at the time of infection, we also observed a significant decrease in body weight with HFD pre fed mice (86%) and an increase in body weight with RD pre-fed mice (112%) at 35 dpi compared to their respective uninfected mice (100%). This appeared to be due to edema which was seen in infected RD pre-fed mice, but which did not occur in infected HFD pre-fed mice. However, uninfected HFD mice tended toward obesity (25% greater body weight) compared to uninfected RD fed mice at d35pi.

### HFD decreased myocardial damage during acute infection

**Figure 2:**
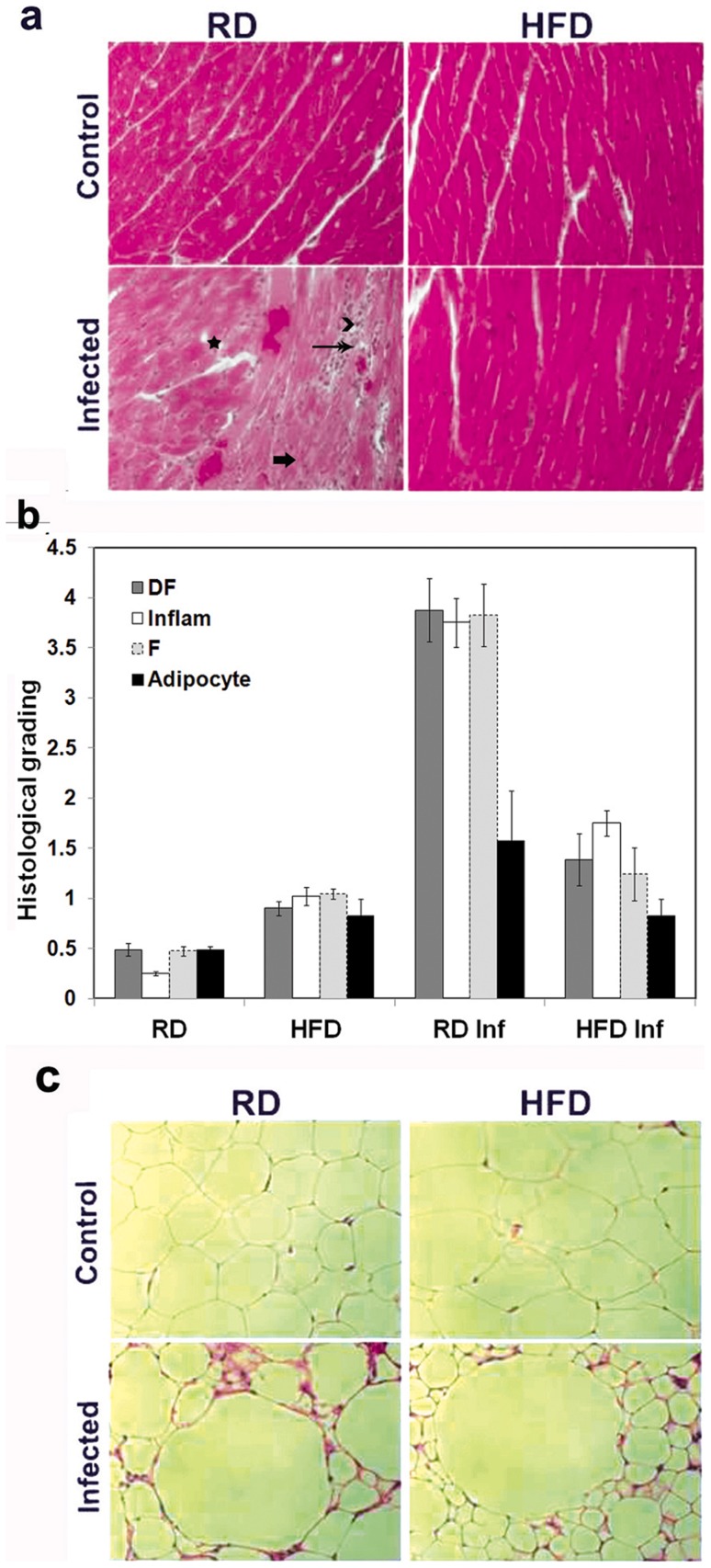
Histology of the myocardium and WAT of mice during acute infection (n = 5, minimum five images/section were analyzed). (**a**) H&E staining displayed significantly more damage in infected RD fed mice hearts compared to the hearts of infected HFD fed mice (degenerating cardiac muscle fibre – black star, inflammation –long black double arrow, fibrosis – thick short arrow, presence of adipocytes or lipid granules - black arrow head) (n = 5). No significant difference between HFD and RD fed uninfected mice hearts was observed (n = 5). Additional images are presented as supplemental Figure 1. (**b**) Histological grading of myocarditis and inflammation was carried out according to experimental groups and classified in terms of degree of degenerating cardiac muscle fibers (DF), inflammation (Inflam), fibrosis (F) and presence of adipocytes (lipid granules). Each class was graded on a five point scale ranging from 0 to 4+ as discussed in Method section, and presented as a bar graph. A dramatic decrease in the severity of the infection (the sum of grading) was observed in the hearts of HFD-fed mice (5.2±0.815) compared with RD fed mice (13±1.367). Bars represent mean values of the data with Standard Error of the mean (SEM) as vertical lines. (**c**) H&E staining displayed slightly enlarged (1.5 fold) and lipid enriched adipocytes in uninfected HFD fed mice compared to the uninfected RD fed mice (top panel). WAT from the uninfected HFD-fed mice displayed slightly enlarged (280±25 µm) and lipid enriched adipocytes compared to WAT of uninfected RD-fed mice (170±60 µm (Fig. 2b, top). WAT from both the infected RD- and HFD-fed mice demonstrated enlarged adipocytes (2.0–2.5 fold enlarged) surrounded by adipocytes with smaller lipid droplets. The average size of inflamed adipocytes of HFD fed mice was 500±150 µm and RD fed mice 400±125 µm during infection. HFD-fed mice had lipid droplets that were significantly smaller compared to RD-fed mice (70±20 µm and 150±30 µm respectively,). There was an increase in the number of dead cells in the WAT of RD-fed mice (Fig. 2b). The number of dead cells were 680±72 and 206±40 in RD fed and HFD fed infected WAT respectively surrounding each inflamed adipocytes (for n = 200 adipocytes) (± SEM).

There were no significant histological differences in the hearts obtained from HFD and RD fed uninfected mice (Fig. 2a). In contrast, the hearts obtained from infected RD fed mice displayed an intense inflammatory reaction most prominent at the right ventricle and left ventricle junction associated with vasculitis and fibrosis. Hearts obtained from infected mice fed a HFD had fewer parasites and a reduction in inflammation and fibrosis (Fig. 2a). Histological scoring of hearts ranged from 0 to 4+ in each of the categories of degenerating cardiac fibers, inflammation, fibrosis and presence of adipocytes. Infected RD animals had higher scores than infected HD fed animals (figure 2b). Summing all scores (maximum cumulative score 16) using this method, the hearts from infected RD fed scored 13 and the hearts from HFD fed infected mice 5.2.

### HFD regulates adipogenesis in *T. cruzi* infected mice during acute infection

Lipolysis and adipolysis are observed during acute *T. cruzi* infection [8], [9]. Adipogenesis is regulated by adipokines such as adiponectin, leptin, PPAR-γ, and TNF-α [11], [12]. These adipokines contribute to the regulation of fatty acid oxidation. The protein encoded by the gene Adig (adipogenin) has been implicated in adipocyte differentiation and fat accumulation.

Examination of the cellular morphology of WAT of infected RD- and HFD-fed mice demonstrated that WAT from the uninfected HFD-fed mice displayed enlarged (280±25 µm) and lipid enriched adipocytes compared to WAT of uninfected RD-fed mice (170±60 µm (Fig. 2b, top). WAT from both the infected RD- and HFD-fed mice demonstrated enlarged adipocytes (2.0–2.5 fold enlarged) surrounded by adipocytes with smaller lipid droplets. The average size of inflamed adipocytes of HFD fed mice was 500±150 µm and RD fed mice 400±125 µm during infection. HFD-fed mice had lipid droplets that were significantly smaller compared to RD-fed mice (70±20 µm and 150±30 µm respectively,). There was an increase in the number of dead cells in the WAT of RD-fed mice (Fig. 2c). The number of dead cells was 680±72 and 206±40 in RD fed and HFD fed infected WAT respectively surrounding inflamed adipocytes (for n = 200 adipocytes). The fat loss in WAT was determined using Oil red O staining [9]. RD-fed infected mice displayed 60% fat loss and HFD-fed infected mice 20% compared to their respective uninfected control mice (p<0.05). qPCR demonstrated a significant decrease in the mRNA levels of the genes involved in adipogenesis such as adiponectin, (−11.5 fold), Peroxisome proliferator activated receptor gamma (PPAR-γ, −6.0), Adipogenin (Adig, −3.5 fold) and leptin (−55 fold) in WAT of RD-fed mice compared to HFD-fed mice during acute (d35pi) infection (Table 1). There was an upregulation of adipogenic genes, except for PPAR-γ, in the heart of infected HFD-fed mice compared with RD-fed mice. These data suggest that HFD increases adipogenesis during infection and that this alteration affects the course of acute *T. cruzi* infection. It is likely that adipogenesis or lipolysis directly affects serum lipid homeostasis.

**Table 1:** Fold change (Mean ± SEM) in the mRNA levels of adipogenic genes in WAT and heart *T. cruzi* infected mice fed on a regular or high fat diet (n = 5).

Gene list	White adipose tissue		Heart	
	RD Infected	HFD Infected	RD Infected	HFD Infected
Leptin	62±5	−7±0.8	1±0.3	8±0.9
Pparg	−6±0.8	1±0.02	2±0.7	2.±0.2
Ppara	−7±0.9	2±0.2	−2±0.8	1±0.04
Adig	−5±0.5	−1±0.18	1±0.5	3±0.3
Adiponectin	−14±1.2	−2±0.6	2.5±0.9	7±0.8

### *T. cruzi* infection alters host lipid homeostasis

**Figure 3:**
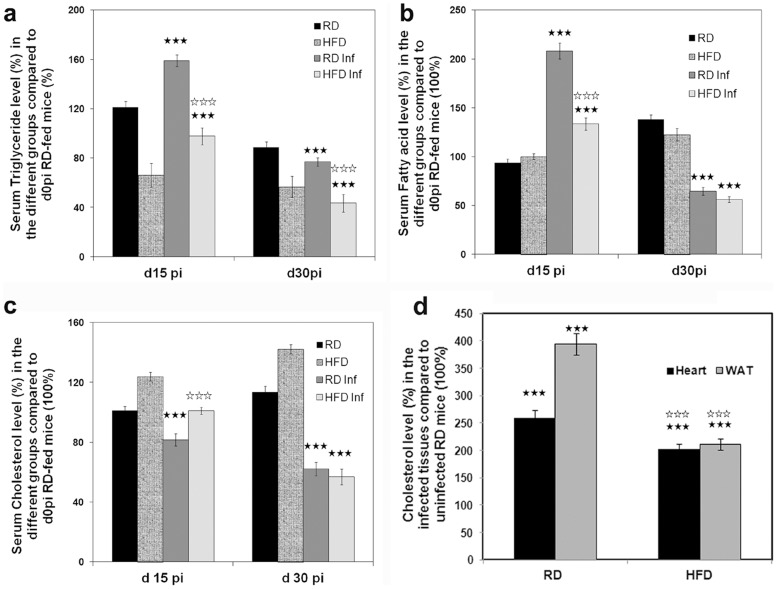
*T. cruzi* infection alters host blood lipid profile and cardiac cholesterol level. (**a**) Both RD and HFD fed infected mice display a significant (p≤0.005) increase in serum TG content at 15 dpi which decrease significantly by the end of acute infection (30 dpi) compared with their respective uninfected groups as quantified by colorimetric analysis. However, serum TG content of uninfected RD fed mice was higher (38%) than uninfected HFD fed mice (n = 5). Results are presented as % difference compared to uninfected RD fed mice at day 0 post infection(d0 pi = 100%). (**b**) Colorimetric analysis of free fatty acids (FFA) demonstrated a significant (p≤0.005) increase in serum FFA content at 15 dpi which decrease significantly by the end of acute infection (30 dpi) compared with their respective uninfected groups (n = 5). Results are presented as % difference compared to uninfected RD fed mice at day 0 post infection(d0 pi = 100%). (**c**) Analysis of total cholesterol (TC) levels in the RD fed mice showed a significant decrease (p≤0.005) even at 15 dpi compared to uninfected mice (n = 5). The uninfected and infected HFD fed mice showed higher levels of TC at 15 dpi compared to uninfected RD and infected RD fed mice respectively, (n = 5, p≤0.005). However at d30pi, both the HFD and RD fed infected mice displayed significantly reduced serum TC levels (p≤0.005). Results are presented as % difference compared to uninfected RD fed mice at day 0 post infection(d0 pi = 100%). (**d**) Acute infection results in elevated LDL/cholesterol levels in heart and WAT. Colorimetric analysis demonstrated a significant (p≤0.005) increase in the cholesterol levels in the hearts (260%) and the WATs (394%) of the infected RD fed mice compared to uninfected RD fed mice (100%) (n = 5). Though infection increased cholesterol levels in the HFD fed infected mice, the analysis demonstrated a significant decrease in their level in both the hearts (202%) and WATs (211%) compared to uninfected RD fed control mice (n = 5). Results are presented as % difference compared to uninfected RD fed mice (100%) (p≤0.005). Bars represent mean values of the data with SEM as vertical lines. Significant change is represented by black stars compared with uninfected RD mice. Significant difference between infected RD and infected HFD is represented by white stars. (p<0.005).

*T. cruzi* has a high affinity for LDL and HDL, and the rate of invasion increases in presence of lipoproteins [7]. We have reported that there is a decrease in serum TG and TC during acute infection in mice fed with a standard chow diet (17% fat) [6]. To analyze whether the HFD would improve the serum lipid levels during infection, we measured TG, FFA and TC levels in HFD fed mice and compared it with RD fed mice at different time points of infection. Serum levels of TG, FFA and TC were significantly decreased in *T. cruzi* infected mice irrespective of the type of the diet fed (Fig. 3). Serum TG content of uninfected RD fed mice was higher (38%) than uninfected HFD fed mice; however the TC was significantly higher (25%) in the uninfected HFD fed mice compared to uninfected RD fed mice. Such alterations in the serum lipid level could potentially regulate myocardial lipid levels and the associated inflammation.

### Acute infection results in elevated LDL/cholesterol levels in tissue

**Figure 4:**
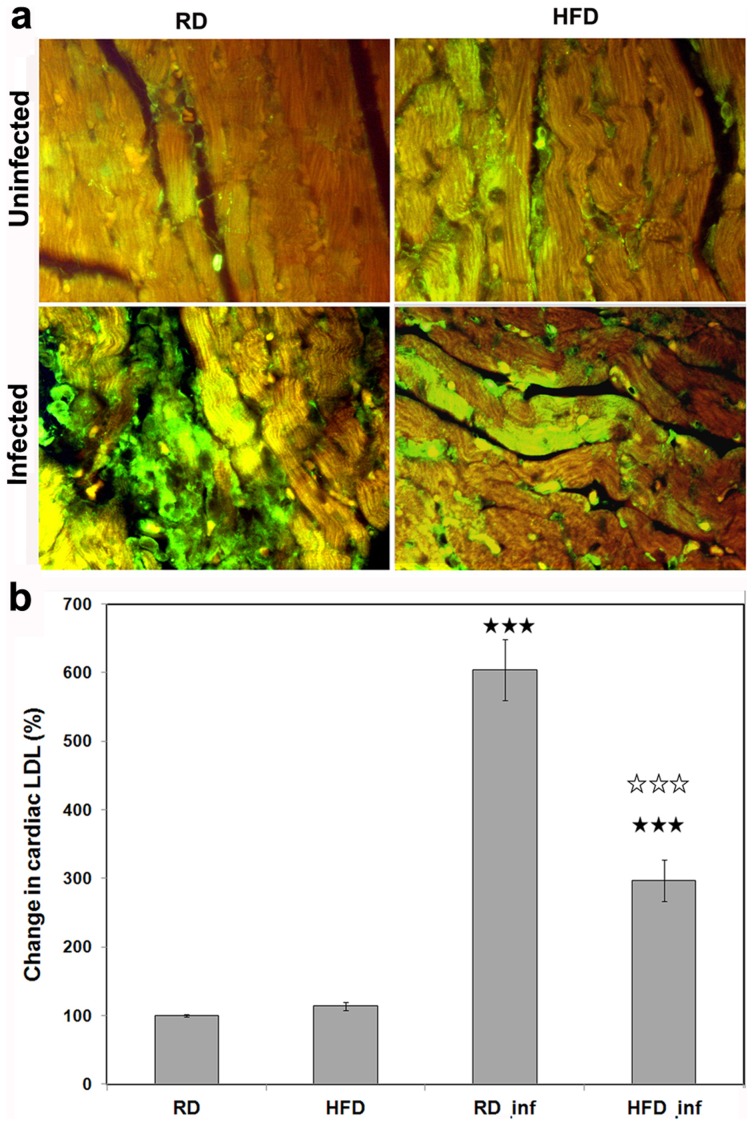
HFD reduces cardiac LDL accumulation during acute infection. (**a**) Immunofluorescence analysis demonstrated a significant increase (p≤0.005) in the accumulation of LDL in the hearts of infected RD fed mice compared with infected HFD mice and their respective controls (n = 5). (**b**) The cardiac LDL levels of the IFA images were quantified and represented as bar graph. Five images from each section (number of mice 5) were quantified using NIH image-J program. Bars represent mean values of the data with SEM as vertical lines. Significant change (p≤0.005) is represented by black stars compared with uninfected RD mice. Significant differences (p≤0.005) between infected RD and infected HFD is represented by white stars.

The rate of *T. cruzi* invasion has been shown to depend on serum cholesterol levels [7]. Invasion results in elevated intracellular/tissue cholesterol levels (Fig. 3d) which could affect triglyceride and cholesterol metabolism (Table 2). Analysis demonstrated a significant increase in the cholesterol levels in the hearts (260%) and the WATs (394%) of the infected RD fed mice compared to uninfected mice (100%). Though infection increased cholesterol levels in the HFD-fed infected mice, this analysis demonstrated a significant decrease in their level in both the hearts (202%) and WAT (211%) compared to uninfected RD-fed control mice. IFA of the myocardium using anti-LDL displayed decreased accumulation of LDL (30%) in infected HFD-fed mice compared to infected RD-fed mice on d35 pi (Fig. 4).

**Table 2:** Infected mice fed on RD and HFD displayed different mRNA levels of the genes involved in lipid metabolism (n = 5; fold change presented as Mean ± SEM).

Gene list	White adipose tissue		Heart	
	RD Infected	HFD Infected	RD Infected	HFD Infected
LDL receptors				
**Cxcl16**	**19±0.3**	**10.0±0.63**	**−103±3.6**	**−470±8.7**
**Stab1**	**67±0.42**	**4.0±0.72**	**19±1.6**	**−1.11±0.02**
**Vldlr**	**−4±0.38**	**−2.0±0.02**	**1.2±0.03**	**−1.09±0.03**
**Ldlr**	**2±0.36**	**1.5±0.10**	**1.3±0.06**	**3.5±0.67**
**Scarf1**	**2.5±0.32**	**2.0±0.06**	**2.0±0.02**	**2.5±0.03**
Apo-lipoproteins				
**Apo a1**	**−165±4.62**	**5±0.92**	**2.5±0.23**	**−1.5±0.03**
**Apo b**	**−54±2.36**	**7±0.78**	**23±2.3**	**5.0±0.96**
**Apo e**	**3.0±0.68**	**1.5±0.03**	**8.3±0.8**	**6.2±0.67**
Triglyceride and fatty acid metabolism				
**Acaa1a**	**−2.5±0.3**	**−1.2±0.3**	**1.44±0.06**	**1.5±0.03**
**Acad9**	**−2.0±0.23**	**2.0±0.06**	**1.07±0.02**	**2.5±0.08**
**Acad10**	**−4.0±0.16**	**1.3±0.3**	**1.20±0.02**	**1.1±0.05**
**Acox1**	**−5.0±0.08**	**−1.2±0.23**	**−2.0±0.05**	**−1.0±0.01**
**Fabp1**	**−30.2±0.8**	**1.70±0.08**	**4.0±0.32**	**10.0±0.6**
**Acsbg1**	**1.5±0.2**	**3.0±0.5**	**3.0±0.56**	**9.0±0.2**
**Lipe**	**−23.0±0.7**	**−1.5±0.5**	**2.0±0.03**	**−1.0±0.1**
Cholesterol metabolism				
**Npc1**	**2.5±0.03**	**3.0±0.8**	**−1.5±0.21**	**1.09±0.32**
**Lcat**	**−2.0±0.08**	**5.0±0.98**	**1.06±0.45**	**2.0±0.08**
**Abca1**	**90±6.2**	**511±72**	**35±4.32**	**32±3.6**
**Abcg1**	**1270±173**	**2.0±0.04**	**2.6±0.03**	**5.5±0.08**
**Cyp39a1**	**1.2±0.03**	**2.8±0.26**	**1.03±0.07**	**−1.4±0.8**
**Cyp7a1**	**−2.5±0.02**	**3.2±0.82**	**1.01±0.02**	**−2.0±0.03**
**Hmgcr**	**4.2±0.20**	**3.5±0.22**	**2.5±0.03**	**5.0±0.23**
**Insig1**	**−6.17±0.87**	**−1.31±0.01**	**−1.5±0.03**	**1.12±0.21**

### Diet differentially regulates inflammatory signaling in WAT and myocardium during acute infection

qPCR analysis demonstrated increased mRNA levels of the inflammatory markers such as TNF-α (5 fold) and IFN-γ (40 fold) in the WAT of HFD-fed mice at d35pi compared to their respective control groups. WAT of RD-fed mice had mostly necrosed cells and showed 2 fold increase in TNF-α and 9.0 fold increase in IFN-γ which was significantly different than HFD-fed mice. The myocardium of the infected RD-fed mice demonstrated a significant increase in TNF-α (92 fold compared to uninfected) whereas, infected HFD-fed mice showed only 28 fold increase in TNF-α. This data supports our histological observations (Fig. 2). The mRNA level of IFN-γ was higher in the myocardium of both the infected HFD and RD-fed mice (185 and 195 fold respectively, compared to their respective controls). There was no statistical difference between the mRNA level of IFN-γ seen in infected HFD and RD fed mice.

### Diet regulates the metabolic state and functioning of the heart during acute infection

**Figure 5:**
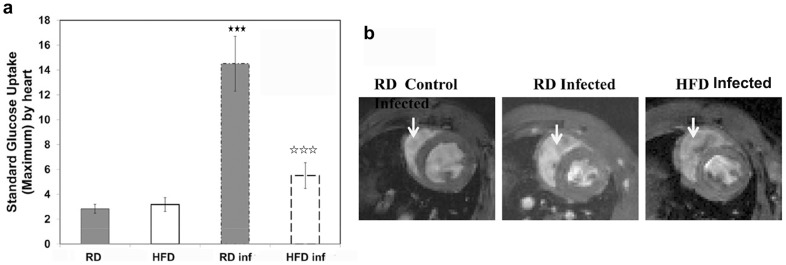
Diet regulates the metabolic state and functioning of the heart during acute infection. (**a**) mPET analysis demonstrated a significant increase in the mean value of the myocardial standard uptake value for the glucose analogue, ^18^F-FDG in the infected RD fed mice compared to HFD infected mice at d30pi (n = 5). Significant change is represented by black stars compared with uninfected RD mice. Significant difference between infected RD and infected HFD is represented by white stars. (p<0.005). (**b**) MRI analysis both at diastole and systole condition showed significantly decrease in the left ventricle internal diameter (LVID) and an increase in the wall thickening of the ventricles during acute infection (both in HFD and RD fed mice) compared with uninfected RD fed mice (arrow – right ventricle) (n = 5). However, the mice fed with HFD showed less abnormalities compared to RD fed mice during infection (n = 5). The right ventricle internal diameter measured was higher for the infected mice and no significant difference was observed between RD and HFD fed mice (Table 3) (n = 5).

Inflammatory cells use glucose as a primary source of metabolic energy, and thus increased uptake of glucose and high rates of glycolysis are characteristics of inflammatory cell infiltration into tissues. Using microPET technology, the metabolic state of the myocardium was assessed by determining the regional uptake of the glucose analogue, ^18^F-FDG. The mean value of the myocardial SUVmax was used to compare the microPET data between the uninfected age-matched controls and infected groups fed on either HFD or RD 30 dpi. Tracer uptake was significantly higher in the myocardium of the RD-fed (14.5±2.2) infected mice compared with infected HFD-fed mice (5.5±1.03) and compared to uninfected control mice (Fig. 5 a). We believe this increased tracer uptake indicates increased inflammation and myocarditis in the RD-fed infected mice [13].

MRI examination of the heart during both diastole and systole revealed a significant decrease in the left ventricle internal diameter (LVID) and an increase in the wall thickening of the ventricles during acute infection compared with uninfected mice (Fig. 5b) (Table 3). The alterations in heart morphology were less pronounced in the infected mice on the HFD compared to those on the RD. The right ventricle internal diameter (RVID) was increased in infected mice and no significant difference was observed between RD- and HFD- fed mice (Table 3). During the acute stage of infection the percent fractional shortening was significantly increased in the infected mice fed RD and likely reflects hypertrophic cardiomyopathy and possibly hypokinetic motion or dyskinesis of the heart wall [14].

**Table 3:** MRI analysis demonstrated altered ventricle diameter, wall thickness and fractional shortening in the hearts of *T. cruzi* infected mice fed on a regular or high fat diet (n = 5; data presented as Mean ± SEM).

Mice	LVID (Diastole)	LVID (Systole)	RVID (Diastole)	RVID (Systole)	Wall average	% Fractional Shortening
RD Control	4.5±0.145	2.2±0.097	2.12±0.09	1.13±0.009	2.4±0.04	49.80±0.55
RD Infected	3.2±0.07	0.8±0.12	2.84±0.1	2.40±0.105	3.5±0.11	74.90±3.19
HFD Control	4.2±0.2	1.7±0.08	1.90±0.09	0.90±0.45	2.0±0.09	61.30±3.07
HFD Infected	3.6±0.011	1.1±0.18	2.90±0.1	2.10±0.029	2.9±0.13	64.29±2.29

(p≤0.005 between RD control and RD infected for LVID, wall average and % Fractional shortening; p≤0.05 between RD infected and HFD infected for LVID, wall average and % Fractional shortening).

Our results demonstrate an alteration in the whole body and systemic lipid homeostasis during acute infection. We further investigated the role of diet on the expression levels of scavenger receptors, lipoproteins, and the proteins involved in lipid metabolism both by qPCR and Immunoblot analysis.

### *T. cruzi* utilizes scavenger receptors for invasion/infection and it is tissue specific during acute infection

*T. cruzi* utilizes the host LDLreceptor to invade cells and invasion upregulates LDLr levels [6], [7]. Analysis of the mRNA levels of other LDL receptors (scavenger receptors), such as stabilin 1 (Stab 1), and scavenger receptor class F member 1 (Scarf 1) in the heart and WAT of both the HFD and RD fed infected mice demonstrated a significant increase compared to their respective uninfected control mice (Table 2). The expression of the oxidized LDL receptor and the chemokine (C-X-C motif) ligand 16 (Cxcl16) was significantly upregulated in WAT of infected mice (Table 2). The fold increase in the expression of Cxcl16 and Stab1 in WAT was 10-fold and 60-fold respectively, in RD-fed infected mice compared with HFD-fed infected mice. Infected heart tissue displayed a significant increase in LDLr and Scarf1 mRNA levels in both RD- and HFD-fed mice, however, a significant increase in the expression of Stab1 was observed only in RD-fed mice (19 fold increase) (Table 2).

### Acute infection differentially regulates the expression of apolipoproteins in heart and WAT

Apolipoproteins play a major role in lipid metabolism and cholesterol homeostasis. We measured the mRNA levels of apolipoproteins such as ApoA, ApoB and ApoE in the heart and WATs of mice fed a HFD and RD during infection. Analysis of the mRNA levels demonstrated a significant difference in the expression levels of these genes in WAT and heart in HFD and RD fed mice (Table 2). RD-fed mice down regulated Apo A1 (−165 fold) and Apo B (−54 fold) expression in WAT and had increased expression of ApoA1 and ApoB in the heart (2 and 23-fold respectively) compared to their respective uninfected mice. In these RD-fed mice Apo E was up regulated both in WAT and heart tissue. Infected HFD mice had increased Apo A1 (5-fold) and Apo B1 (7-fold) in the WAT and increased levels of Apo A1, Apo B1 and Apo E in heart tissue. The expression levels of Apo A1, Apo B1 and Apo E were significantly lower than that seen in RD-fed mice. It is likely that changes in apolioprotein levels affect systemic cholesterol homeostasis in these RD- and HFD-fed mice.

### Altered triglyceride metabolism in heart and WAT during acute infection

Fatty acid (FA) transport and β-oxidation are important in the functioning of WAT and the heart. The genes responsible for FA transport, TG metabolism and β-oxidation such as acetyl-Coenzyme A acyltransferase 1A (Acaa1a), mitochondrial acyl-CoA dehydrogenase family members (Acad 9 &10), acyl-CoA oxidase (Acox1), glycerol-3-phosphate dehydrogenase (Gpd1), FA acid binding protein (Fabp1) and acyl-CoA synthetase bubblegum family member 1 (Acsbg1) were analyzed (Table 2). WAT from the infected RD-fed mice demonstrated a significant fold decrease in the expression of these genes; however infected HFD-mice showed no significant change in the majority of these genes (the only exceptions being Acad9 (2 fold) and Acsbg1 (3 fold) where there was increased expression compared to uninfected control mice). In the hearts of both RD and HFD fed infected mice the levels of these genes were significantly increased especially in HFD compared to control mice (Table 2).

### Acute infection affects cholesterol trafficking, efflux mechanism and metabolism

The Niemann–Pick disease type C1 (Npc1) gene encodes a large protein that resides in the limiting membrane of endosomes and lysosomes and mediates intracellular cholesterol trafficking via binding of cholesterol to its N-terminal domain [15]. Analysis of the mRNA levels demonstrated an elevated expression of Npc1 mRNA (3-fold) in the WAT of both the RD- and HFD-fed infected mice. No significant difference, however, was seen in Npc1 in the hearts of these mice. The gene lecithin-cholesterol acyltransferase (LCat) encodes the extracellular cholesterol esterifying enzyme Lcat, which is required for cholesterol esterification [14] is down regulated in infected RD-fed mice (7 fold) compared to the infected HFD-fed mice (Table 2). The esterification of cholesterol is required for cholesterol transport [16]. We investigated the expression of genes involved in cholesterol efflux like ATP binding cassette (Abc) transporters such as Abca1 and Abcg1 in WAT and heart [17]. With cholesterol as their substrate, these proteins function as cholesterol efflux pumps in the cellular lipid removal pathway. Abca1 is significantly increased in both the WAT and heart of infected mice (Table 2). Abcg1 is mainly associated with macrophage cholesterol efflux. Abcg1 is significantly upregulated (1270 fold) in the WAT of infected RD fed mice compared with infected HFD fed mice which suggests that increased macrophage infiltration is associated with RD-fed mice. We have published that there is an increased influx of macrophages in WAT during acute infection [8], [9]. Abcg1 is significantly higher in the hearts of both HFD- and RD-fed infected mice (Table 2). Intracellular cholesterol is mainly converted to bile acids in liver. Cytochrome p450 monooxygenases like Cyp39a1 and Cyp7a1 were up regulated (3- and 5-fold respectively) in WAT of HFD-fed infected mice compared with infected RD-fed mice (Table 2). Cyp39a1 and Cyp7a1are endoplasmic reticulum proteins involved in the conversion of cholesterol to bile acids [18], [19]. Cyp7a1 is the rate limiting enzyme in the primary pathway of bile acid synthesis [19]. Even though adipocytes are not a classical bile acid synthesizing tissue, it has been shown that farnesoid x-receptor (FXR) a nuclear receptor which is involved in bile acid synthesis is expressed in adipose tissue during metabolic dysfunction [20]. The mRNA levels of HMG co A reductase (Hmgcr), a rate limiting enzyme in the cholesterol biosynthesis is upregulated in both the WAT and the hearts of both RD-and HFD-fed mice during acute infection (Table 2).

### Immunoblot analysis confirmed *T. cruzi* infection induced systemic alterations in lipid/cholesterol homeostasis

**Figure 6:**
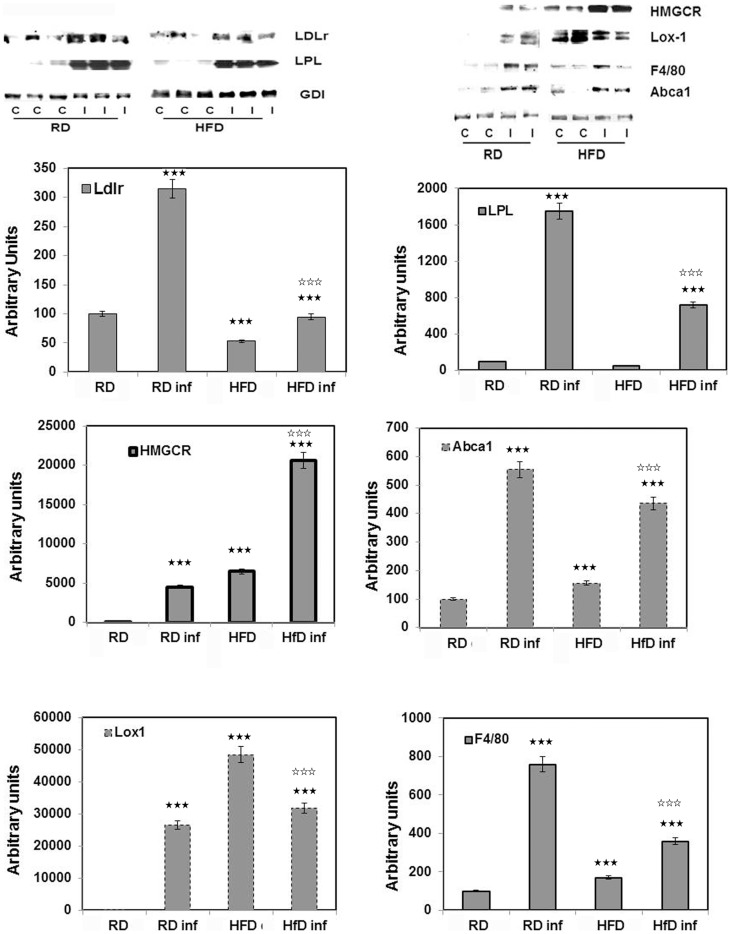
Immunoblot analysis of the hearts confirmed *T. cruzi* infection induced systemic alterations in lipid/cholesterol homeostasis and macrophage infiltration. Protein levels of LDLr and oxidized LDLr (LOX1), LPL, Abca1 and HMGCR were altered in the hearts during *T. cruzi* infection. Macrophage infiltration marker (F4/80) was significantly increased in the infected RD fed mice compared to infected HFD fed mice. Fold change in the protein levels were normalized to GDI expression and represented as bar graph (n = 5, p≤0.05 (two black stars) to ≤0.005 (three black stars). Bars represent mean values of the data with SEM as vertical lines. Significant change is represented by black stars compared with uninfected RD mice. Significant difference between infected RD and infected HFD is represented by white stars (p≤0.005).

**Figure 7:**
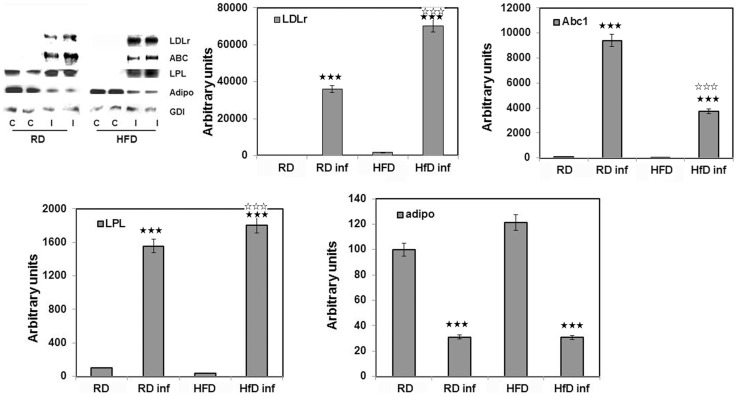
Immunoblot analysis of the WAT confirmed *T. cruzi* infection induced systemic alterations in lipid/cholesterol homeostasis. WAT from both the RD and HFD fed infected mice showed upregulated LDLr, Abca1 and LPL activity. Unlike hearts from the infected mice WAT showed no multimer adiponectin and also significantly reduced adiponectin monomers. Fold change in the protein levels were normalized to GDI expression and represented as bar graph (n = 5, p≤0.005). Bars represent mean values of the data with SEM as vertical lines. Significant change is represented by black stars compared with uninfected RD mice. Significant difference between infected RD and infected HFD is represented by white stars (p≤0.005).

Immunoblot analysis demonstrated an upregulation of LDLr in infected mice. LDLr levels in the hearts of infected RD-fed mice were significantly higher compared to that of infected HFD-mice (Fig. 6). In WAT of infected mice the reverse was seen (Fig. 7). We also analyzed the expression of LOX1 (oxidized LDLr) in heart tissue and found results similar to that seen with LDLr in RD and HFD fed infected mice (Fig. 6). Hearts from infected mice displayed increased macrophage infiltration (probed with anti-F4/80), lipoprotein lipase activity (anti-LPL) and cholesterol efflux levels (ant-Abca1) (Fig. 6). However, the expression levels of F4/80 (−2 fold), LPL (−2.5 fold) and Abca1 (−0.7 fold) were lower in the infected HFD mice compared with the infected RD mice.

**Figure 8:**
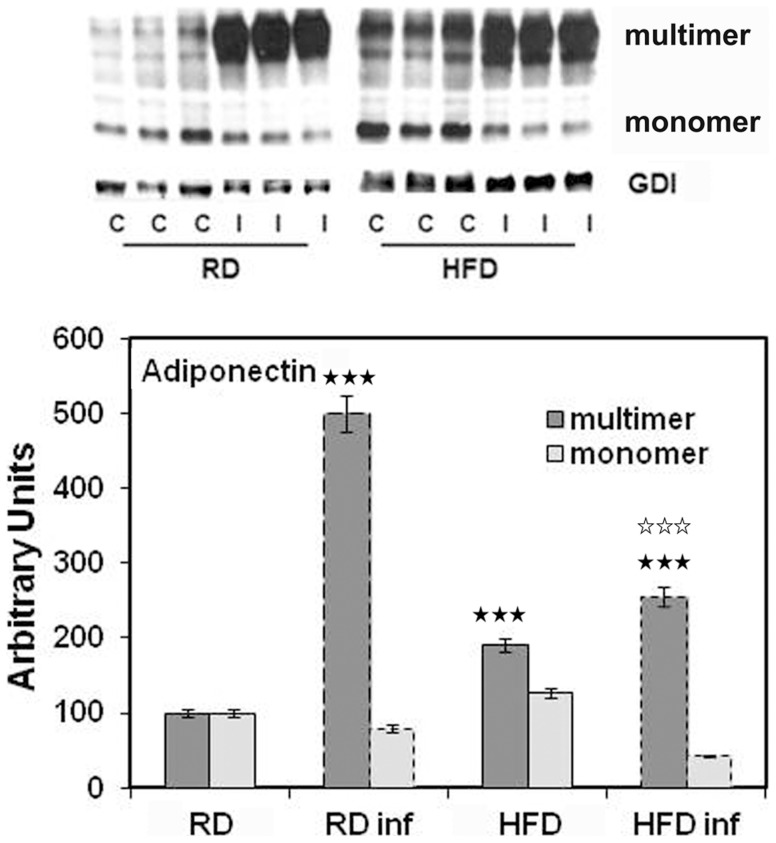
Immunoblot analysis of the hearts demonstrated altered adiponectin levels during acute *T. cruzi* infection. Immunoblot analysis demonstrated increased expression of adiponectin (multimer) in the hearts of infected mice compared to uninfected mice both in RD and HFD fed mice. Adiponectin monomer levels were significantly decreased in the hearts of infected mice. However, immunoblot analysis demonstrated significantly different levels of adiponectin levels between HFD and RD fed mice both at uninfected and infected conditions. Fold change in the protein levels were normalized to GDI expression and represented as bar graph (n = 5, p≤0.005 for both monomer and multimer). Bars represent mean values of the data with SEM as vertical lines. Significant change is represented by black stars compared with uninfected RD mice. Significant difference between infected RD and infected HFD is represented by white stars (p≤0.005).

The enzyme involved in the rate limiting step of cholesterol biosynthesis HMGCR is significantly increased (400-fold) in the hearts of infected mice. In the hearts of uninfected HFD-fed mice there was an increase in HMGCR compared to uninfected RD- fed mice. This suggests that HFD induces HMGCR but that infection further increases the expression of HMGCR in heart tissue (Fig. 6). Interestingly, infected heart tissue had increased amount of adiponectin multimers, but decreased monomers (Fig. 8), whereas we observed no multimers in infected WAT. Also the amount of adiponectin monomers were decreased in infected WAT (Fig. 7 and Fig. 8). Uninfected HFD fed mice heart tissue had higher adiponectin levels compared to uninfected RD fed mice hearts which was similar to what was seen in WAT from these mice (Fig. 7 and Fig. 8).

## Discussion

*T. cruzi* has a high affinity for lipoproteins/cholesterol [7] and depends on host lipid molecules for invasion and survival [6], [7], [21]. Adipose tissue is the largest endocrine organ in the body and is a rich source of lipids and is involved in energy homeostasis. Previously we reported that adipose tissue is an early target of *T. cruzi* infection and serves as a reservoir for parasites [8], [9]. *T. cruzi* infection-induced lipolysis is a hallmark of acute infection [8], [9] and lipolysis is known to alter lipid homeostasis. Diet plays a major role in adipogenesis and in lipid homeostasis. In the present study, we systematically analyzed the impact of high fat (HFD, 60% fat) and regular diet (RD, 10% fat) on an acute model of Chagas disease and demonstrated a link between diet, adipogenesis and myocarditis. HFD increased adipogenesity and reduced lipolysis which affected peripheral parasitemia and parasite load in the heart. Even though a significant difference in the parasite load of heart was demonstrated between the RD- and HFD- fed infected mice, the adipose tissue of HFD fed mice had a significantly higher number of parasites. We believe the increased fat tissue in HFD mice resulted in a sequestration of parasites (i.e. a sponging effect) that may have led to a reduction in the parasite load in the heart. Consistent with this hypothesis, HFD-fed mice displayed an increased survival rate (95%) with diminished myocardial damage during acute infection compared with RD-fed mice. HFD induced a modest obese condition (as seen with uninfected mice) may bring metabolic changes during infection. Overall, this increase in survival with HFD supports the “so-called” obesity paradox hypothesis [21].

The various techniques we employed to investigate cardiac structure and function clearly demonstrated that in HFD-fed mice there was a significant amelioration of myocardial dysfunction compared to RD-fed mice. This is likely due to the role of adipogenesis and lipolysis during infection. Lipolysis is a characteristic marker of acute infection in mice where there is a significant decrease in total body fat between d10 and d35d pi. HFD-fed mice increased adipogenesis and reduced the rate of adipocyte lipolysis compared to RD-fed mice. The parasite load in the WAT was higher in these HFD mice compared to RD. The pro-inflammatory marker TNF-α was higher in the WAT of HFD-fed mice at this time point of infection. Adipocyte lipolysis was significantly higher in the infected RD-fed mice as indicated by serum triglyceride and fatty acid levels. Overall, this change in fatty acid metabolism probably contributes to increased parasitemia in RD-fed mice and a higher parasite load in heart.

Serum cholesterol levels were reduced with a concomitant increase in intra-organelle LDL/cholesterol levels (Fig. 3). We have demonstrated the accumulation of LDL/cholesterol in adipose tissue and the hearts [22] during infection. Accumulated lipids need to be degraded (through lipases) before undergoing further degradation through the β-oxidation pathway. Increased lipolysis activation through LPL was observed both in the hearts and WAT of infected mice. There was a significant increase in the LPL expression in the hearts of RD-fed mice compared with HFD-fed mice suggesting that more lipids accumulated in RD-fed mice due to increased lipolysis and increased parasite load. Hormone sensitive lipase (Lipe) is significantly down regulated only in the WAT of RD-fed mice. No significant change was observed in the hearts of either RD- or HFD-fed mice at d35pi.

FA transport and β-oxidation are important signaling pathways in the functioning of WAT and heart. White adipocytes are not mitochondrial rich cells, unlike brown adipocytes which are mitochondrial rich, and the elevation of infection induced lipids may cause a burden on mitochondrial oxidative capacity in WAT leading to necrosis. qPCR demonstrated a down-regulation of the mRNA levels of many of the genes involved in triglyceride and FA metabolism which reflects the oxidative state in WAT of RD-fed mice. Previously, we reported a significant loss of WAT during acute infection [8], [9]. The heart, however, could sustain this load as it is rich in mitochondria and we observed increased FA and triglyceride metabolism both in the RD- and HFD-fed mice. Interestingly, it has been recently reported that host FA metabolism is essential for the persistence of *T. cruzi* amastigotes [23]. Decreased expression of Insig1, a regulator of cholesterol biosynthesis through SREBP in both the hearts and WAT of RD fed mice is also responsible for an increased HMGCR expression and cholesterol biosynthesis.

*De novo* cholesterol biosynthesis is highly regulated and depends on intracellular cholesterol levels [24]. When there is a depletion in intracellular cholesterol, cells respond with a SREBP-activated increase in HMGCR and LDLr expression which results in endogenous cholesterol biosynthesis and LDL-mediated uptake of cholesterol. In the present study, we found increased SREBP levels and increased HMGCR and LDLr protein levels in the tissues when the cells already had elevated intracellular LDL/cholesterol levels due to parasite invasion suggesting that infection results in dysfunctional cholesterol homeostasis in tissues and changes in the regulation of these key cholesterol homeostasis genes.

Expression of the ABC transporters is highly upregulated during macrophage differentiation and cholesterol loading, and they synergize to mediate cholesterol transport to Apo-A1 [25]. The expression of these genes was significantly altered during infection.

Adiponectin, an adipokine secreted by the WAT, plays an important role in regulating glucose and lipid metabolism and controlling energy homeostasis in insulin-sensitive tissues [26]. Adiponectin is considerably reduced in the WAT, but, importantly produced in the heart during acute infection. Previously, we demonstrated a reduction in serum adiponectin levels at 15 days post infection (dpi) returning to normal by 30 dpi [8], [9]. WAT (fat cells) significantly decreases during infection and thus, the amount of WAT in infection may not be sufficient to maintain normal serum adiponectin. As the adiponectin multimers are functionally active, further studies are required to confirm that the heart secretes adiponectin during *T. cruzi* infection, the mechanism by which this occurs and the significance of elevated adiponectin levels in the heart during acute infection.

Lipogenic and adipogenic markers such as leptin, adiponectin, adipogenin and PPAR are greatly reduced in the WAT of RD-fed mice compared to HFD-fed mice, but significantly increased in the heart especially in the HFD-fed mice. This suggests that there are tissue specific of acute *T. cruzi* infection related to the altered lipolysis and lipogenic status of different tissues. Previously, we demonstrated the role of host LDLr in *T. cruzi* invasion [6]. qPCR analysis revealed (Table 2) the association of other LDL receptors and modified LDL receptors such as classical LDLr, very low density lipoprotein receptor (VLDLr), STAB1, CXCL16 and SCARF1 in the hearts and WAT during acute infection. The significant increase and the decrease in the LDLr protein levels in the WAT and heart respectively, of the HFD-fed infected mice compared with RD-fed mice, suggests that WAT of HFD-fed mice and the hearts of RD-fed mice are targets of *T. cruzi* during the late phase of the acute infection. Thus, an increased parasite load in the hearts of RD-fed mice resulted in elevated cardiac LDL/cholesterol levels, macrophage infiltration (F4/80 staining) and inflammation (TNF-α) compared with HFD-fed mice. Overall, it is clear that HFD modulates the effect of *T. cruzi* infection on myocarditis and mortality in this acute model and this was confirmed by microPET and MRI analyses.

HFD is known to alter the metabolic state of the host leading to diabetes and obesity. HFD induces the metabolic syndrome and this alteration in the host affects the pathogenesis of acute Chagas disease resulting in a decreased heart parasite burden and an increased survival rate. This observation in the mouse model is consistent with the “obesity paradox” that has been described for some chronic infections reflecting an observation that obesity can have a positive effect on disease outcome. To this end, the metabolic syndrome may have evolved as a response to “times of plenty” when the extra calories could be used for metabolic changes that would allows the host to better deal with chronic infectious diseases such as *T. cruzi* [21]. Herein, we report, for the first time on alterations in the lipid signaling net work due to diet, adipogenesis, and lipolysis in the setting of acute *T. cruzi* infection. We believe that these alterations contribute to the pathogenesis of acute Chagas disease. The rate of survival and the severity of the myocardial damage are related to the adipogenic and the lipolytic status of adipose tissue and the heart. Lipid and cholesterol homeostasis is completely altered by the infection which warrants further mechanistic studies to understand the pathogenic role of LDL/cholesterol in the progression of Chagasic cardiomyopathy which can now be considered, in part, to be a lipidopathy (onset of cardiomyopathy due to abnormal intracellular lipid level).

## Supporting Information

Figure S1 **Histology of the myocardium of mice during acute infection (n = 5).** H&E staining displayed significantly more inflammation and damage in infected RD fed mice hearts compared to the hearts of infected HFD fed mice.
(TIF)
