# Supplementary figures and images for: High Fat Diet Modulates Trypanosoma cruzi Infection Associated Myocarditis
Source: PLoS Negl Trop Dis. 2014 Oct 2;8(10):e3118. doi: 10.1371/journal.pntd.0003118 (PMC4183439; doi:10.1371/journal.pntd.0003118)

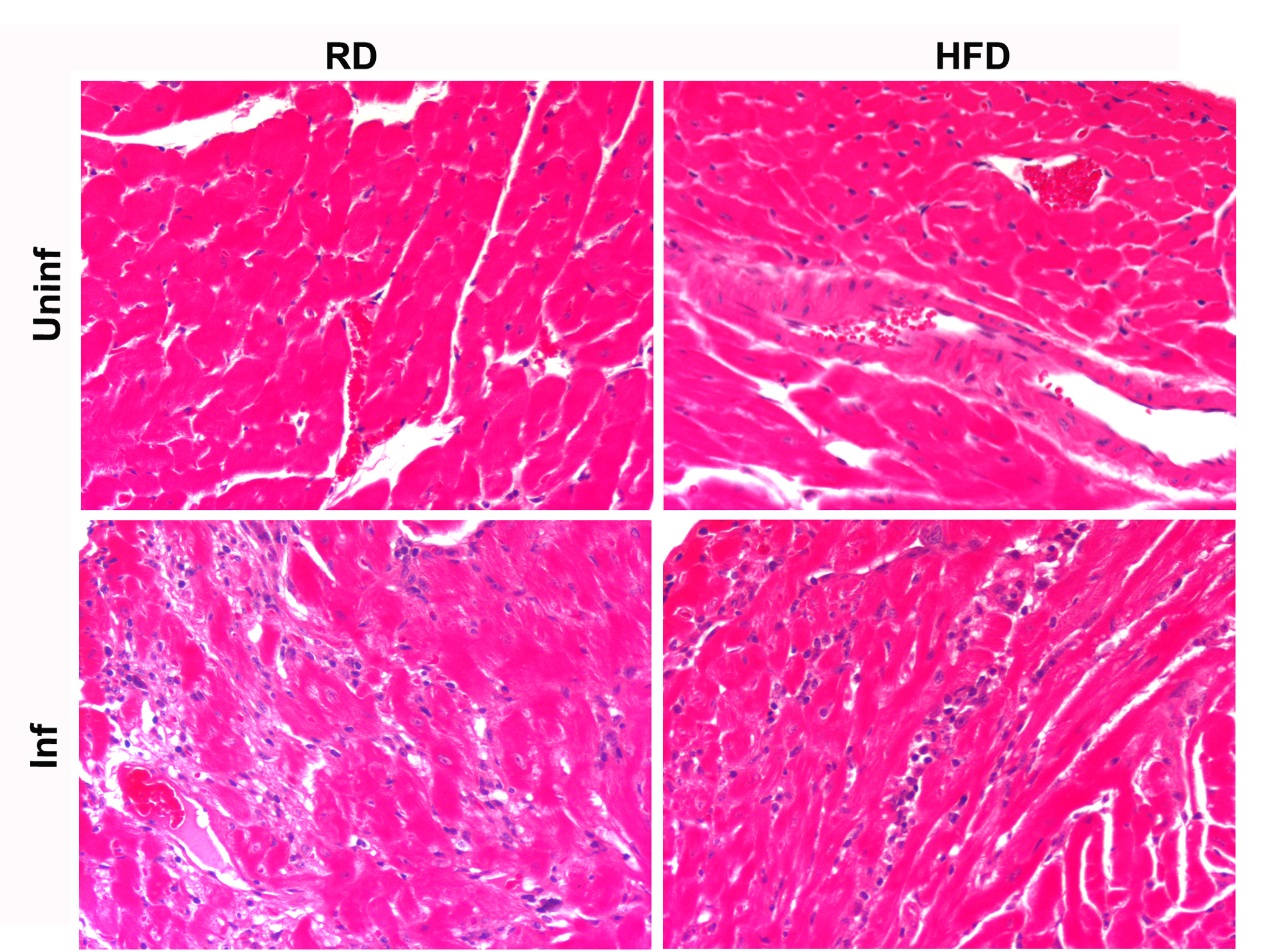

Supplement: Figure S1 — Histology of the myocardium of mice during acute infection (n = 5). H&E staining displayed significantly more inflammation and damage in infected RD fed mice hearts compared to the hearts of infected HFD fed mice. (TIF) [file pntd.0003118.s001.tif]
